# Special Issue “Construction Materials and Other Related Materials: Basic Theory, Applied Technology and Advanced Research Methods (2nd Edition)”

**DOI:** 10.3390/ma18184339

**Published:** 2025-09-16

**Authors:** Xi Du, Youliang Chen, Qinglong You, Rafig Azzam, Tomas Manuel Fernandez-Steeger, Bin Peng

**Affiliations:** 1 Department of Civil Engineering, University of Shanghai for Science and Technology, Shanghai 200093, China; 2 Rheinisch-Westfälische Technische Hochschule Aachen, 52056 Aachen, Germany; 3 Institut für Angewandte Geowissenschaften, Technische Universität Berlin, 10587 Berlin, Germany

## 1. Introduction

The advancement of construction materials science requires integrating fundamental theory, practical applications, and advanced research methodologies [1,2,3,4,5]. This Special Issue demonstrates this multidisciplinary approach through diverse investigations across materials and engineering contexts [6].

Theoretical foundations are established through studies of material properties and behavior, including lightweight concrete with sintered aggregates, carbonation curing processes with activated magnesium oxide, and the geotechnical behavior of lateritic soils. These investigations expand our understanding of both traditional and innovative construction materials.

Advanced research methodologies showcase the evolution of analytical techniques. Machine learning approaches for predicting asphalt content represent the integration of artificial intelligence in pavement engineering. Numerical simulations of expansive soft rock behavior demonstrate computational methods for complex geotechnical challenges, while systematic reviews of structural stiffness effects highlight meta-analytical approaches in knowledge synthesis.

Practical applications bridge theory and implementation through experimental analyses of airport concrete pavements under real loading conditions and studies of early-age masonry properties addressing construction safety.

A classification scheme was developed according to the research approaches and methodological focus of the publications. The papers can be categorized into two primary groups:

(1) Theoretical and Experimental Studies, which focused on fundamental material characterization, laboratory investigations, and computational modeling of construction materials including lightweight concrete properties, carbonation curing mechanisms, masonry compressive behavior, soft rock mechanical response, and structural vibration analysis;

(2) Applied Engineering and Field Implementation, which emphasized practical applications, field performance assessments, and innovative design methodologies for infrastructure projects such as tropical soil utilization in road construction, machine learning-based asphalt mixture optimization, and airport pavement system evaluation under operational conditions.

This categorization distinguishes between research that advances fundamental understanding of material behavior and studies that directly address real-world engineering challenges and implementation strategies.

## 2. Short Description of the Articles Presented in This Special Issue

The first part of this Special Issue investigates the short-term and long-term mechanical properties of lightweight concrete incorporating sintered fly ash aggregate. Based on two concrete mix designs with different water-to-binder ratios, key mechanical parameters including the secant modulus of elasticity, cylindrical strength, cubic strength, axial tensile strength, splitting tensile strength, flexural strength, and shrinkage and creep strains were systematically determined using standard methods and specialized equipment. The results demonstrated that this lightweight concrete could achieve high strength grades, with short-term mechanical properties being generally consistent with previous research findings. However, the shrinkage and creep values were significantly higher than those reported in prior literature. This important finding indicates that long-term performance parameters must be accurately tested using standardized methods and appropriate equipment, as non-standard approaches may lead to unreliable results. This study provides an essential design parameter basis for the engineering application of this environmentally friendly lightweight concrete utilizing sintered aggregates derived from waste fly ash in prestressed structures.

The second contribution focuses on developing a novel lightweight carbonated solidification slurry material from the perspective of resource utilization and carbon sequestration, employing reactive magnesium oxide, slag powder, and carbide slag as stabilizers, with carbonation reactions induced through CO_2_ foaming. The study systematically analyzed the influence patterns of reactive magnesium oxide content, CO_2_ foam dosage, and stabilizer content on the physical and mechanical properties of the material, including wet density, flowability, water content, and unconfined compressive strength. Results showed that wet density increased with increasing magnesium oxide and stabilizer contents but decreased with increasing CO_2_ foam dosage. Flowability decreased with increasing magnesium oxide and stabilizer contents, while increasing CO_2_ foam dosage improved flowability. Unconfined compressive strength increased with curing age and stabilizer content but decreased with increasing CO_2_ foam dosage, and long-term curing significantly enhanced both water content and compressive strength of the material. This research proposed a sustainable carbon utilization solidification strategy that achieves waste resource utilization using low-carbon cementitious materials and industrial wastes, providing a theoretical foundation and engineering reference for the development of novel low-carbon cementitious materials and the application of carbonation solidification technology in soil stabilization and lightweight concrete fields.

Another study presented examines purple clay from the São Paulo region of Brazil, which demonstrated good potential for pavement engineering applications, challenging the conservative evaluation of traditional soil classification methods. Through repeated load triaxial tests, X-ray diffraction, and scanning electron microscopy, the study systematically evaluated the permanent deformation and resilient modulus characteristics of this lateritic clay. Results showed that high concentrations of iron oxides in the material significantly enhanced soil cohesion and mechanical strength. When compacted under intermediate Proctor compaction energy, its resilient modulus values were comparable to certain granular materials reported in literature, demonstrating good potential as pavement material. However, under high stress levels, its permanent deformation was considerably higher than reference materials, indicating the significant influence of loading conditions on material behavior. The research also found that traditional USCS and HRB classification systems often underestimate the mechanical potential of fine-grained lateritic soils, while the MCT classification method could more accurately reflect the engineering characteristics of such tropical soils. This study provides a scientific basis for the rational utilization of lateritic materials in road engineering and holds important significance for expanding the application of abundant and low-cost lateritic resources in infrastructure construction in tropical regions.

The fourth research explores the application of machine learning methods to optimize Marshall mix design for asphalt mixtures. The study compares traditional methods with various machine learning algorithms (including linear regression, support vector regression, decision tree, random forest, gradient boosting, K-neighbors, and neural networks) for predicting key performance parameters of asphalt mixtures. Through an analysis of 60 datasets covering granite and limestone aggregates with different gradations, the research found that neural network models perform best in predicting Marshall stability, flow, air voids, and other parameters, achieving the highest R^2^ values and the lowest mean squared errors. The results demonstrate that compared with conventional experimental methods, machine learning, particularly neural network technology, can more accurately predict optimal asphalt content and mixture performance, not only improving prediction accuracy but also significantly reducing testing time and costs, providing a data-driven solution for asphalt pavement material design and sustainable infrastructure development.

The fifth investigation conducted an experimental study on Airport Precast Concrete Pavement (APCP) performance at Gimpo International Airport in Seoul, Korea, implementing a novel design concept that reduced slab thickness by 30 mm while incorporating reinforcing bars to maintain structural integrity. Through comprehensive field measurements using temperature gauges, strain gauges, and displacement transducers, the researchers analyzed APCP behavior under both environmental loads (temperature variations) and moving airplane loads (B737 and A320 aircraft). Key findings revealed that the APCP exhibited typical concrete pavement characteristics including pronounced curling behavior, with strains during daytime significantly larger than those during nighttime due to slab curling effects, and a maximum vertical deformation of approximately 0.48 mm within acceptable ranges for concrete pavements. The study demonstrated that this reinforced APCP design, which eliminates the need for complete asphalt layer removal and base reconditioning, maintains structural performance while reducing construction time and costs. Using various fatigue failure analysis models including S-N curve, Darter, Aas-Jakobsen, and Roesler equations, the researchers predicted a service life exceeding 30 years even under the most conservative assumptions, concluding that this innovative APCP approach can be successfully applied to airport pavement rehabilitation projects.

The sixth paper investigates the development patterns of compressive properties in newly constructed masonry structures during the curing process through experimental studies of early-age mortar cubes and masonry prisms. The study finds that the compressive strength of mortar is extremely low in the initial casting period and increases logarithmically with curing time, with failure modes showing significant differences before and after the onset of the primary cement hydration phase—early stages exhibit plastic compression deformation, while later stages present typical conical shear failure. For masonry prisms, although the compressive failure load has little correlation with curing time from a life-safety perspective, from a performance standpoint, early-age masonry shows clear compressive bearing limitations to prevent excessive deformation of mortar joints. This research provides important material property foundations for the design of temporary bracing systems in masonry construction, helping to improve construction safety guidelines and scheduling for concrete block masonry.

The seventh work employs COMSOL Multiphysics 6.1 numerical simulations to investigate moisture absorption dynamics and mechanical responses during tunnel excavation in expansive soft rock, with comparative analysis of various strength criteria and non-associated flow rules. The study demonstrated that the Mohr-Coulomb criterion more effectively restricts water-induced swelling in expansive soft rock compared to the Drucker-Prager criterion, with the combination of the Mohr-Coulomb criterion and Drucker-Prager compression meridian providing the strictest limitations on rock expansion, resulting in reduced horizontal displacement and ground uplift while increasing displacement at the tunnel’s bottom arch and decreasing it at the top arch. Furthermore, the research revealed significant impacts of shear dilation angle, burial depth, and support resistance on surrounding rock stress and displacement fields: increased shear dilation angles correlated with greater rock expansion and surface deformation; deeper burial depths led to stabilized horizontal displacement but increased ground uplift; and the presence of support resistance created circular high-stress zones while significantly affecting the relative displacement distribution between the tunnel’s bottom and top arches, providing valuable theoretical foundations for support system design in expansive soft rock tunnels.

The eighth part presents a systematic literature review that takes an in-depth look at the relationship between structural stiffness and vibration periods in concrete buildings, showing that increasing the stiffness of key structural components—beams, columns, and shear walls—can effectively shorten building vibration periods, which in turn indicates a faster structural response to external forces. The research team compares the strengths and limitations of different analytical methods, including finite element analysis and modal analysis, while revealing a nonlinear relationship between building height and vibration periods, and it emphasizes that in earthquake-prone regions a rational configuration of rigid elements such as shear walls can significantly enhance seismic performance and reduce the risk of seismic damage. At the same time, the study identifies limitations in existing theoretical models, notably the simplified treatment of boundary conditions, assumptions of material homogeneity, and insufficient consideration of variations in soil conditions, and, based on these findings, it recommends fully leveraging emerging technologies such as artificial intelligence and machine learning, adopting comprehensive multivariable analytical approaches, and developing more comprehensive predictive models to optimize the seismic design of concrete buildings.

## 3. Conclusions

This Special Issue brings together diverse research outcomes in the field of construction materials, spanning from fundamental theory to applied technology and advanced methodologies. These studies involve various materials including lightweight concrete, recycled aggregates, and lateritic soils, reflecting the construction industry’s exploratory efforts toward sustainable development.

In terms of research methodologies, the studies combine traditional experimental approaches with cutting-edge technologies such as machine learning, numerical simulation, and systematic analysis. While addressing practical engineering challenges including infrastructure durability, seismic performance, and construction safety, researchers have also given full consideration to environmental protection factors.
